# Effects of Hot Sterilization on Torsional Properties of Endodontic Instruments: Systematic Review with Meta-Analysis

**DOI:** 10.3390/ma12132190

**Published:** 2019-07-08

**Authors:** Mario Dioguardi, Diego Sovereto, Riccardo Aiuto, Luigi Laino, Gaetano Illuzzi, Enrica Laneve, Bruna Raddato, Vito Carlo Alberto Caponio, Antonio Dioguardi, Khrystyna Zhurakivska, Giuseppe Troiano, Lorenzo Lo Muzio

**Affiliations:** 1 Department of Clinical and Experimental Medicine, University of Foggia, Via Rovelli 50, 71122 Foggia, Italy; 2 Department of Biomedical, Surgical, and Dental Science, University of Milan, 20122 Milan, Italy; 3 Multidisciplinary Department of Medical-Surgical and Odontostomatological Specialties, University of Campania “Luigi Vanvitelli”, 80121 Naples, Italy

**Keywords:** torsional fatigue, endodontics, NiTi alloy, endodontic instruments, torsional resistance, flexural fatigue, autoclave, sterilization, mechanical property, fatigue properties

## Abstract

Sterilization is a fundamental step in the reuse of endodontic instruments. The sterilization procedure involves disinfection, cleaning, washing, drying, packaging, and sterilization by heat. Heat sterilization can lead to changes in the physical and mechanical properties of dental instruments. These changes can affect the external surfaces via micropitting, corrosion, a reduction in cutting capacity, and/or an influence on the resistance to cyclic fatigue or to torsional fatigue. In this study, we examined the modification of the torsional properties of endodontic instruments after hot sterilization, and compared the properties with instruments not subjected to hot sterilization cycles in terms of resistance to torsional fatigue and deflection angle in NiTi and steel instruments. The following work was performed based on the PRISMA indications. Studies were identified through bibliographic research using electronic databases. A total of 725 records were identified in the PubMed and Scopus databases. A total of 685 records remained after exclusion by year of publication (1979 to 2019). With the application of the eligibility criteria (all articles pertaining to the issue of sterilization in endodontics), we found 146 articles, which decreased to 130 articles after elimination of duplications. There were 45 articles that studied the influences of sterilization procedures on the physical and mechanical characteristics of the instruments, and 12 that measured parameters related to resistance to torsional fatigue. Applying the inclusion and exclusion criteria resulted in a total of eight articles for quantitative analysis. The meta-analysis results show a pejorative effect of torsional fatigue for NiTi instruments subjected to heat sterilization compared to the non-sterilized control.

## 1. Introduction

Sterilization is a fundamental step in the reuse of endodontic instruments. The sterilization procedure follows the steps of disinfection, cleaning, washing, drying, packaging, and sterilization by heat [1]. The disinfection and cleaning steps reduce the bacterial load and remove debris from the blades of the instruments, and then the sterilization step kills any form of microorganism, including spores [2]. The most widely used method described in the scientific literature for sterilization in the dental field is heat sterilization. Sterilization by heat involves the use of autoclaves that reach a temperature of 134 °C, which, with the action of steam at a pressure of 30 psi, sterilizes the instruments [3].

Heat sterilization can lead to changes in the physical and mechanical properties of dental instruments. These changes can affect the external surfaces with micropitting and corrosion phenomena [4], a reduction in cutting capacity, and/or by influencing the resistance to cyclic fatigue or torsional fatigue [5].

An unfavorable event that can occur during the preflaring, shaping [6], and probing phases is rupture of the endodontic instrument [7]. Ruptures can be caused by cyclic fatigue stress or torsional fatigue stress. Rupture due to torsion occurs when the instrument is blocked in the channel, losing its cutting capacity. The alloy can undergo a deformation that is first elastic and then plastic, and due to fatigue, it breaks [8].

Heat sterilization procedures can influence the torsional properties of endodontic instruments. Studies on resistance to torsional stress have related the number of sterilization cycles to the torsional properties, showing variations in the resistance to torsion and in the angle of deflection during torsion. In 2011, King et al. [9] reported a reduction in torsion resistance for GT Series X rotary instruments (Dentsply, Tulsa Dental Specialities, Tulsa, OK, USA) and an increase in resistance for Twisted Files (SybronEndo, Orange, CA, USA). Additionally, in 2011, Casper et al. [10] showed an increase in resistance to torsional fatigue for controlled memory (CM) wire files, whereas Hilt in 2000 reported no influence on the torsional properties of both steel and nickel-titanium alloy (NiTi) instruments [11].

The challenge is that sterilization cycles can either lead to deterioration, have no influence, or improve the mechanical properties of the instruments. The most recent study on the subject was conducted by Alazemi et al., in 2014, which showed a recovery effect of the instrument shape after autoclave sterilization [12]. The scientific literature is therefore not always in agreement about the effects of sterilization. These differences may be derived from the heterogeneity of the instruments investigated. The various endodontic instruments produced by various brands differ not only in diameter and taper at the tip, but also in the shapes of the sections and the characteristics of the alloys.

The aim of this review was to try to understand these apparent controversies between the various studies with the help of systematic methodology and meta-analysis. We investigated the influences of heat sterilization on torsional properties by differentiating instruments between steel and NiTi alloys. Systematic reviews, where the relationship between torsional fatigue and sterilization procedures were investigated, were not conducted until 2019. The literature, however, includes updated revisions on sterilization procedures in the endodontic field and systematic reviews on cyclical fatigue, but they are never related to each other [1,13]. The results of this review could therefore help endodontists follow a more accurate approach to the reuse of endodontic files, and understand the effects of autoclaving on instruments.

We paid attention, using a qualitative analysis of the studies, to the variations in the torsional fatigue resistances of M wire, CM wire and Electrical Discharge Machining (EDM) alloys of endodontic instruments, following sterilization by heat. We aimed to meet the needs of modern, constantly-evolving endodontics and provide useful information on new alloys available to the clinician.

## 2. Materials and Methods 

The following work was performed based on the PRISMA indications [14]. The population, intervention, comparison, and outcome (PICO) questions of our study were endodontic instruments, modification of hot sterilization on the torsional properties of endodontic instruments, endodontic instruments not subject to hot sterilization cycles, and resistance to torsional fatigue and deflection angle for NiTi and steel instruments, respectively. The research question based on the PICO approach was how hot sterilization affects the torsional characteristics of NiTi and steel endodontic instruments compared to the same non-sterilized instruments.

After a screening phase, the eligible articles were qualitatively analyzed to investigate the effects of sterilization procedures on the resistance to torsional fatigue of endodontic instruments used for probing, glide path, and shaping of the endodontic canal.

### 2.1. Eligibility Criteria

In this study, we considered literature reviews, in vitro studies, and clinical studies concerning the theme of sterilization and the influence of the sterilization on the mechanical properties of endodontic instruments, which were conducted in recent years and published with abstracts in English. We decided to choose articles from the last 40 years (1979 to 2019) because the disinfection and sterilization procedures changed radically after infectious contaminants were discovered, such as the HIV and HCV viruses and the prion of spongiform encephalopathy. The methods used to manufacture the instruments changed with the introduction of new alloys and new instruments.

The articles considered potentially eligible were those that discussed the influences of sterilization and disinfection procedures on the physical and mechanical characteristics of endodontic instruments. The potentially eligible articles were finally subjected to a full-text analysis to verify the use for a qualitative and quantitative analysis.

The inclusion criteria applied to the quantitative analysis included all studies that discussed the sterilization methods of endodontic instruments. The exclusion criteria included all studies that did not report data on resistance to torsional fatigue or on the angle of deflection due to torsion, of NiTi and steel endodontic instruments sterilized by heat. We also excluded the articles not in English, and those prior to 1979.

### 2.2. Research Methodology

Studies were identified through bibliographic research on electronic databases [15]. The literature search was conducted on the search engines PubMed and Scopus. The search was conducted between 13 January 2019 and 20 February 2019, and the last search for a partial update of the literature was conducted on 17 May 2019. The following search terms were used on PubMed and Scopus: “Endodontic sterilization” PubMed 326, Scopus 236; “endodontic autoclave” PubMed 37, Scopus 50; “cyclic fatigue” AND “sterilization” PubMed 19; and “torsional” AND “sterilization” PubMed 30 (Table 1).

### 2.3. Screening Methodology

The records obtained were subsequently screened by two independent reviewers (M.D. and G.I.), and a third reviewer (G.T.) acted as a decision maker in doubtful situations. The screening included analysis of the title and the abstract to eliminate records not related to the themes of the review. After the screening phase, the overlaps were removed and the full texts of the articles were analyzed, from which those eligible for qualitative analysis and inclusion in the meta-analysis for the four outcomes were identified. The outcomes sought by the two reviewers were:

(1) Primary outcome: Variation in torsional fatigue resistance for NiTi endodontic instruments compared to the control not subjected to hot sterilization;
(2) Secondary outcome: Variation in the flexion angle for NiTi endodontic instruments compared to the control not subjected to hot sterilization;
(3) Tertiary outcome: Variation in resistance to torsional fatigue for steel endodontic instruments compared to controls not subjected to hot sterilization;
(4) Quaternary outcome: Variation in the flexion angle for steel endodontic instruments with respect to the control not subjected to hot sterilization.

The fourth reviewer, with supervisory duties, was L.Lo.M. The K agreement between the two screening reviewers was 0.655 (Table 2). The K agreement was based on the formulas in the Cochrane Handbook for Systematic Reviews [16].

The Newcastle-Ottawa scale for case-control studies was used to assess the risk of bias in the included studies [17]. The quantitative analysis was performed with the Rev Manager software 5.3 (Cochrane collaboration, Copenhagen, Denmark).

### 2.4. Data Analysis

The statistical analysis of the data was performed using the Rev Manager 5.3 software (Copenhagen, 153 Denmark, The Nordic Cochrane Centre, The Nordic Cochrane Collaboration, 2014) and the results were represented by forest plots for each of the outcomes.

**Figure 1:**
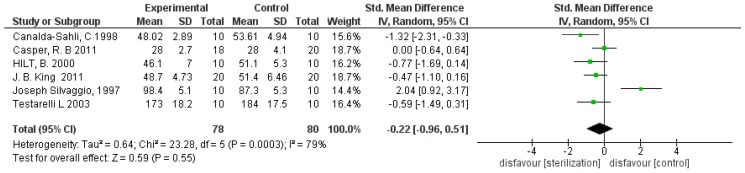
Forest plot of the random effects model of the meta-analysis of the primary outcome.

For the primary outcome, the variation in resistance to torsional fatigue was compared following hot sterilization of NiTi instruments by measuring the torsional moment (g·cm) compared with the non-sterilized controls using a torquemeter. The comparison shows high heterogeneity of the studies, with an *I*^2^ equal to 79%. For this reason, a random effects model was used. Overall, for the primary outcome, the meta-analysis is in favor of non-sterilized NiTi instruments (control). The studies that presented data with a statistically significant difference are: Silvaggio et al. [18] in favor of the control and Canalda-Sahli [19] against the group subject to sterilization. Casper [10] is exactly at the center of the non-effect line, whereas the remaining three studies are unfavorable for the group subjected to heat sterilization even if their confidence intervals intercept the non-effect line (Figure 1).

**Figure 2:**
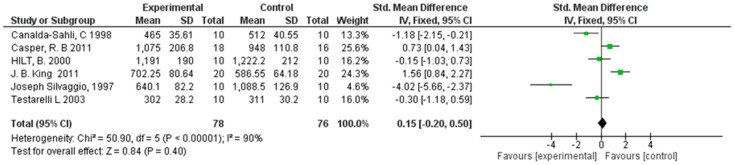
Forest plot of the random effects model of the meta-analysis of the secondary outcome.

For the secondary outcome, the variation in the angle of deflection (degrees) up to instrument rupture was compared in the NiTi instruments between the control group and the sterilization group using a torsimeter. The comparison showed high heterogeneity among the studies, with an *I*^2^ equal to 90%. For this reason, for the second outcome, a random effects model was applied so as to not minimize the roles of smaller-dimension studies. For the second outcome, the forest plot is in favor of the subject group compared to the control. The studies that reported statistically significant data in favor of the experimental group are Silvaggio et al. [17] and Canalda-Sahli et al. 1998 [19], whereas those in favor of the control group are Casper et al. 2011 [10] and King et al. 2011 [9] (Figure 2).

**Figure 3:**
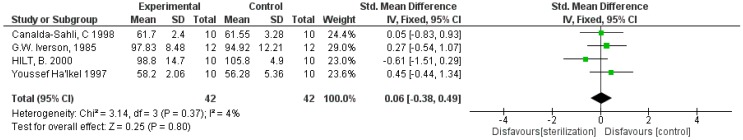
Forest plot of the fixed effects model of the meta-analysis of the tertiary outcome.

For the tertiary outcome, the variation in resistance to torsional fatigue was compared following hot sterilization of steel instruments, measuring the torsional moment (g·cm) with respect to the non-sterilized controls using a torquemeter. The comparison showed low heterogeneity between the studies, with an *I*^2^ of 4%, and a fixed effects model was applied. For the tertiary outcome, the forest plot is in a position of no effect for the two groups. None of the included studies presented statistically significant data (Figure 3).

**Figure 4:**
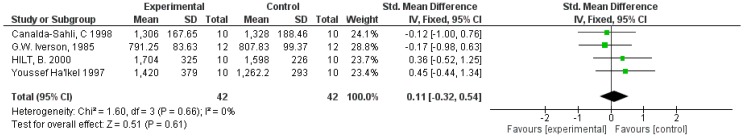
Forest plot of the fixed effects model of the meta-analysis of the quaternary outcome.

For the quaternary outcome, the variation in the angle of deflection (degrees) up to instrument failure, measured using a torquemeter, in steel instruments between the control group and the sterilization group was compared. The comparison shows low heterogeneity between the studies, with an *I*^2^ of 0%, and a fixed effects model was applied. The forest plot has a slightly shifted graph in favor of the control group, but with low significance. None of the studies reported statistically significant data in favor of the control or of the group subjected to sterilization (Figure 4).

## 3. Results

A total of 725 records were identified on the PubMed and Scopus databases (Table 1). After screening the articles with restriction by year of publication (1979 to 2019), there were 685 records. With the application of the eligibility criteria (all the articles pertaining to the issue of sterilization in endodontics), there were 146 articles. There were 130 articles after eliminating overlaps. There were 45 articles that discussed the influences of the sterilization procedures on the physical and mechanical characteristics of the instruments, and 12 that measured parameters on resistance to torsional fatigue.

**Figure 5:**
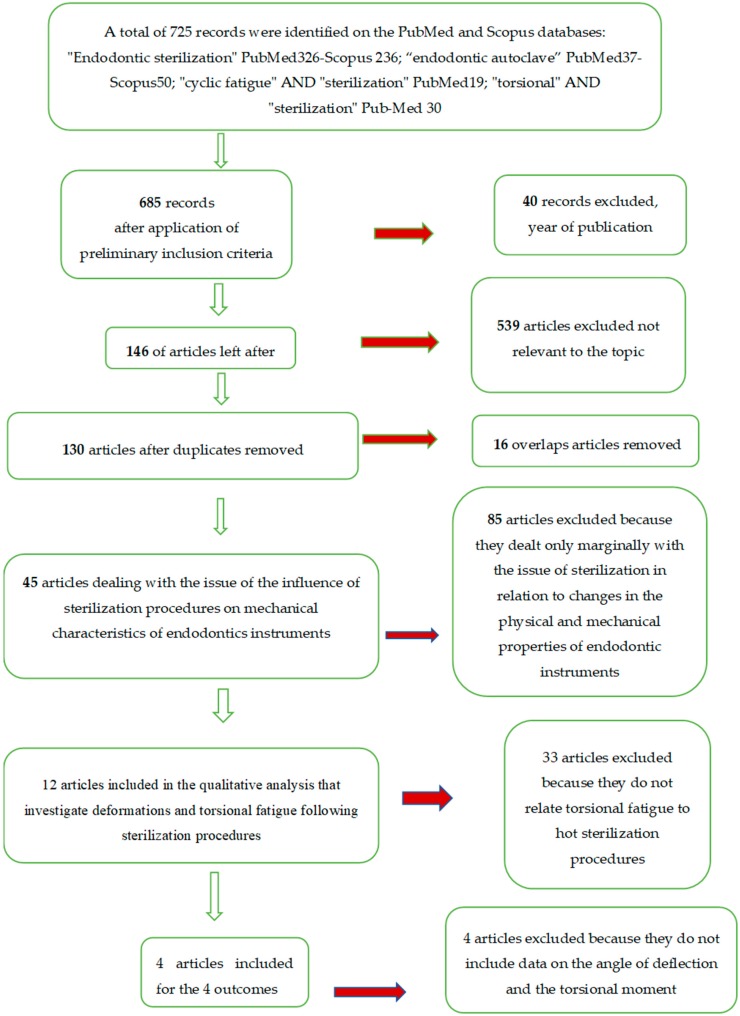
Flow chart of the different phases of the systematic review.

Applying the inclusion and exclusion criteria resulted in a total of eight articles for quantitative analysis. Six articles were in reference to the primary and secondary outcomes, and four to the tertiary and quaternary outcomes. The whole selection and screening procedures are described in the flow chart (Figure 5).

### 3.1. Study Characteristics and Data Extraction

The included studies for quantitative analysis were:

- First and second outcomes: Hilt et al. 2000 [11], Silvaggio et al. 1997 [18], Canalda-Sahli et al. 1998 [19], King 2011 et al. [9], Casper et al. 2011 [10] and Testarelli et al. 2003 [20];
- Third and fourth outcomes: Iverson et al. 1985 [21], Hilt et al 2000 [11], Canalda-Sahli et al. 1998 [19] and Haikel et al. 1997 [22].

The extracted data included the magazine (author, data, and journal), the endodontic instrumentation object of measurement (name, taper, and diameter at tip), the method of sterilization by heat (temperature, pressure, and time), the type of instrumentation used for the measurement, and the data related to torsional fatigue (torsional moment, deflection angle, bending moment, and Knoop hardness). The data extracted for the four outcomes are shown in Table 3 and Table 4.

We extracted the data concerning the torsional moments and the deflection angles of the new alloys (M wire, CM, and Twisted Files) present in the market since 2010 and which hold considerable interest for the endodontist. The studies that analyzed the heat sterilization and the torsional properties of the M wire and CM alloy instruments were King et al. [9] and Casper et al. [10] (Table 5).

### 3.2. Risk of Bias

The risk of bias was assessed through the Newcastle-Ottawa case-control scale. The results are reported in detail in Table 6 for each category, a value of one to three was assigned (1 = low and 3 = high).

The risk of bias within the individual studies was low enough that the methods of investigation adopted for the controls (not sterilized with heat) were identical to the cases (sterilized with heat) included in the meta-analysis. For Silvaggio et al. [18] only, we note an unclear assignment of cases and controls for all the experiments performed in the study.

The risk of bias between the various studies was considered high, and partly limited the importance of the results. The heterogeneity between the studies is highlighted by three main factors: Diversity of the endodontic instruments used (only similar in taper and diameter at the tip), different apparatuses for measuring the torsional moment and the angle of deflection, and inconsistency in the method of hot sterilization.

**Figure 6:**
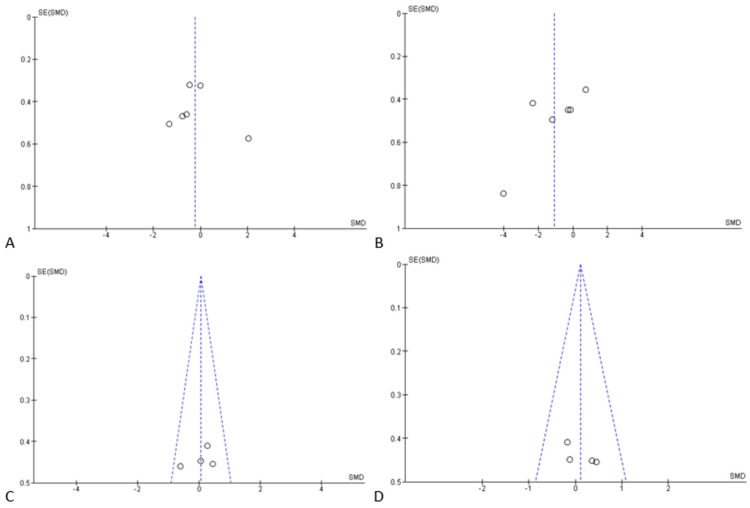
Funnel plots of the evaluation of heterogeneity for the (**A**) first, (**B**) second, (**C**) third, and (**D**) fourth outcomes.

The heterogeneity of the studies is represented by funnel plots of the four outcomes in Figure 6.

## 4. Discussion

The scientific literature provides contradictory findings about the effects of heat sterilization on the torsional properties of NiTi and steel instruments used in endodontics.

The three most studied parameters in the literature for measuring torsional fatigue are: (1) The torsional moment (g-cm), which represents the force necessary to break the instrument; (2) the deflection angle (degrees), which represents the instrument’s torsion angle at breaking point; and (3) the bending that represents the necessary force (g-cm) to bend an endodontic instrument at a defined angle.

In the literature, the studies reporting an improvement in the resistance to torsional fatigue for endodontic instruments are:

(1) Iverson [21], which reported a statistically significant increase in fatigue resistance only for the burn Unifile (De Trey, Bois Colombes, France) instruments after 10 sterilization cycles;
(2) Silvaggio [18], which reported an increase in torsional fatigue resistance for profile instruments (Tulsa Dental Product Tulsa, OK), but not for all sizes;
(3) Canalda-Sahli [19], which reported an increase in flexibility for titanium instruments and a decrease/increase for NiTi instruments;
(4) King [9], which reported improvements in resistance to torsional fatigue for Twisted Files instruments and a reduction for the GT Series X M wire after only three sterilization cycles by autoclaving;
(5) Casper et al. [10], which reported an increase in resistance to torsional fatigue after heat sterilization for CM wire, with a recovery effect of the shape after sterilization; and
(6) Alazemi et al. [12], which, like Casper et al. [10], reported a recovery effect for HyFlex CM instruments (Coltène-Whaledent, Altstätten, Switzerland).

Only one study included in the quantitative analysis reported statistically significant data for the GT Series X, whereas the remaining articles reported data with non-significant differences, showing no effect of the sterilization procedures on the torsional characteristics of endodontic instruments. The studies that showed increased resistance to torsional fatigue were mainly conducted on NiTi instruments. King et al. [9] reported an increase for Twisted Files. These instruments are built following a twist, and undergo thermal heating and cooling processes that stabilize the NiTi alloy. The thermal heating induced by hot sterilization make it even more stable, increasing resistance to torsional fatigue, which is also valid for M wire alloys.

We obtained additional data from the meta-analysis. For the first outcome, we found an unfavorable effect of sterilization (10 cycles of autoclaving) on resistance to torsional fatigue in NiTi instruments, but with low statistical significance.

The second outcome concerns the variation in the deflection angle (up to failure) following torsion. In this case, the data from the meta-analysis were significant, with a reduction in the deflection angle in the group subjected to heat sterilization. This indicates a greater stability of the austenitic and martensitic phases of the NiTi alloy following heat treatment during sterilization.

For the tertiary and quaternary outcomes, with the meta-analysis we established that there is no change in the resistance to torsional fatigue, nor is there an improvement in the steel tools with hot sterilization procedures in a pejorative sense, according to Hilt et al. [11] and Canalda-Sahli et al. 1998 [19].

### New Alloys: M Wire, CM, and EDM—Qualitative Analysis of the Studies

Since 2009, the development of new alloys has led to the construction of new endodontic instruments with improved physical and mechanical characteristics, including increased resistances to cyclic fatigue and torsional fatigue. The new alloys that have been developed are M wire, CM wire, and EDM alloys [23].

M wire alloys were introduced in 2009, and are characterized by a more stable martensitic phase, which is reached during clinical use at higher temperatures. This depends on a thermomechanical manufacturing process that makes the martensitic phase less subject to breakage, with an intermediate phase from the austenitic phase, defined as the “R-phase”. M wire alloys have a high-temperature form recovery effect, showing 6% recovery of plastic deformation induced by stress [24]. The endodontic instruments built using M wire alloys are: ProTaper Next (Dentsply Maillefer, Ballaigues, Switzerland) [25], WaveOne Gold (Dentsply Maillefer, Ballaigues, Switzerland), Twisted Files, and the Gt X and Profile Vortex series (Dentsply Tulsa Dental, Tulsa, OK, USA).

CM wire alloys are always constructed with a thermomechanical process, but with shape-memory control.

EDM alloys are manufactured using an electrical discharge machining technique, always with a controlled memory alloy.

There are three articles related to the variations in torsional properties with the sterilization procedures, of which the data from two articles are shown in Table 5. King et al. [9] reported a statistically significant reduction for the M wire alloys, and specifically the GT Series X, after three sterilization cycles (torsional moment 85.67 ± 9.59 g·cm, angular deflector 483.11 ± 66.57 degrees) (Table 5). The study reports, however, a slight increase in resistance to twisting for the other M wire alloy instruments (twisted file). Casper et al. [10] reported no statistically significant alteration in the torsional properties in a pejorative sense for the M wire (Twisted files, ProFile Vortex) alloys and for the CM wire alloy (CM wire file). Alfoqom Alazemi et al. [12] reported recovery of the shape in two-thirds of the cases after hot sterilization in the HyFlex CM wire.

The data reported in the literature for the new M wire, CM wire, and EDM alloys show that they have similar form recovery after heat sterilization and an increase in resistance to torsional fatigue, with some exceptions, such as the GT Series X [26].

The data on the increase in resistance to torsional fatigue are also supported by data on cyclic fatigue resistance. Zhao et al. [27] reported an increase in cyclic fatigue resistance for HyFlex CM instruments, in accordance with the data on torsional fatigue. Hilfer Pet al. [28] reported a reduction in the resistance to cyclic fatigue following repeated cycles of autoclaving for the GT Series X (M wire alloy), which does not affect the M wire alloy instruments.

## 5. Conclusions

From the literature analysis, only 12 scientific articles published in international journals that related heat sterilization procedures to the resistance to torsional fatigue in detail were identified. Of these 12 articles, only eight were included in the meta-analysis for the four outcomes.

From the meta-analysis, we found that heat sterilization procedures reduce the torsional moment of NiTi instruments, with a low significance. When translated into clinical terms, this means a reduction in the resistance to torsional fatigue, with a greater risk of fracture. There is also a reduction in the angle of deflection following rupture for the group subjected to autoclaving. The clinical significance of this is not entirely clear to us, and certainly requires future investigation. For steel instruments, there is no change in the resistance to torsional fatigue, whether it is expressed in terms of torsional moment or deflection angle.

The torsional fatigue data on the newer M wire, CM wire, and EDM alloys are fundamentally important for the endodontics clinician. Awareness of the better reliability of these new instruments following sterilization is beneficial for endodontists who proceed with the sterilization of the instruments before use. The data agree that there is a lower risk of fracture in the case of reuse compared to the old generation instruments.

## Figures and Tables

**Table 1:** Complete overview of the search methodology. Records identified by databases: 725; records selected for qualitative analysis: 12; records selected for quantitative analysis: 8.

Provider Database	Key Words	No. of Records	Number of Records after Restriction by Year of Publication (1979 to 2019)	No. of Articles Remaining after the Elimination of Records Not Related to Sterilization of Endodontic Instruments	No. of Articles after Removing Duplications	No. of Articles Addressing Influence of Sterilization Procedures on Mechanical Characteristics of Endodontic Instruments	No. of Articles Included in The Qualitative Analysis (Influence of Hot Sterilization on Torsional Properties)	No. of Articles Included in the Quantitative Analysis for the Four Outcomes
Pub-med	“Endodontic sterilization”	326	287	35	\	\	\	\
Pub-med	“endodontic autoclave”	37	36	21	\	\	\	\
Pub-med	“cyclic fatigue” AND “sterilization”	19	19	16	\	\	\	\
Pub-Med	“torsional” AND “sterilization”	30	30	17	\	\	\	\
SCOPUS	Endodontic AND Sterilization	263	263	36	\	\	\	\
Scopus	“endodontic” AND” autoclave”	50	50	21	\	\	\	\
Total records		725	685	146	130	45	12	8

**Table 2:** K agreement calculation, Po = 0.86 (Proportion of agreement), Pe = 0.594 (Agreement expected), K agreement = 0.655 (<0 no agreement, 0.0–0.20 slight agreement, 0.21–0.40 fair agreement, 0.41–0.60 moderate agreement, 0.61–0.80 substantial agreement, 0.81–1.00 almost perfect agreement). The K agreement was calculated from the 45 articles to include eight articles with the application of the inclusion and exclusion criteria.

		Reviewer 2	Reviewer 2	Reviewer 2	
		Include	Exclude	Unsure	Total
Reviewer 1	include	8	4	1	13
Reviewer1	exclude	0	31	0	31
Reviewer 1	unsure	1	0	0	1
	total	9	35	1	45

**Table 3:** Extracted data from selected studies (primary outcome and secondary outcome).

Autor, Date, Journal, Reference Number	Endodontic Instruments, Test Instruments	Sterilization Method	No. of Instruments	Temperature, Pressure, Exposure Time	Torsional Moment (g·cm) Mean ± SD	Angular Deflection (degrees) Mean ± SD	Bending Moment Mean ± SD (g·cm)	Knoop Hardness (kg/rnrn^2^)
Hilt. 2000 Journal of Endodontics, [11]	NiTi file (30 size 02 taper), torsional testing apparatus	10 cycles Statim autoclave	10	132 °C (270 °F), 20 psi, 30 min	46.1 ± 7.0	1191 ± 190	\	322 ± 18
		Control	10	\	51.1 ± 5.3	1222. 2± 212	\	330 ± 32
Silvaggio, 1997, journal of endodontics, [18]	Profile taper 4 series 5 (0.279 mm) size, Torquemeter Memocouple.	Dry heat 10 cycles	10	190–204 °C Atmospheric 46 min	98.4 ± 5.1	640.1 ± 82.2	\	\
		control	10	\	87.3 ± 5.0	1088.5 ± 126.9	\	\
Canalda-Sahli, 1998, international endodontic, [19]	Nitiflex (30 size 02 taper), Digital Torquemeter Memocouple, Maillefer	10 cycles autoclave	10	136 °C, 2.2 bar, 10 min	48.02 ± 2.89	465 ± 35.61	17.16 ± 1.81	\
		control	10	\	53.61 ± 4.94	512 ± 40.55	21.57 ± 1.11	\
King, 2011, international endodontic journal, [9]	Twisted Files (25, size 06 taper) Torsiometer/Memocouple	Autoclave statim 7 cycles	20	132 °C 2.2 bar for 6 min,	48.70 ± 4.73	702.25 ± 80.64	\	\
		control	20	\	51.40 ± 6.46	586.55 ± 64.18	\	\
Casper, 2011, journal of endodontics, [10]	Twisted Files (25 size 04 taper), microprocessor-controlled torsiometer	7 cycles Autoclave statim	18–18	132 °C 2.2 bar, for 6 min	28 ± 2.7	1075 ± 206.8	\	\
		control	20–16	\	28 ± 4.1	948 ± 110.8	\	\
Testarelli, 2003 minerva stomatologica, [20]	Hero (size 30 size 02 taper), Torsiometer/Memocouple	10 cycles autoclave	10	124 °C 2.2 bar, for 20 min	173 ± 18.2	302 ± 28.2	147 ± 12.4	\
		control	10	\	184 ± 17.5	311 ± 30.2	146 ± 12.9	\

**Table 4:** Extracted data from selected studies (tertiary outcome and quaternary outcome).

Autor, Date, Journal, Reference Number	Endodontic Instruments, Test Instruments	Sterilization Methods	No. of Instruments	Temperature, Pressure, Exposure Time	Torsional Moment Mean ± SD (g·cm)	Angular Deflection Mean ± SD (Degrees)	Bending Moment Mean ± SD (g·cm)	Knoop Hardness (kg/rnrn^2^)
Iverson, 1985, journal of endodontics, [21]	K flex (35 size 04 taper), torqumeter memocouple	Autoclave 10 cycles	12	127 °C, 30 psi, 30 min	97.83 ± 8.48	791.25 ± 83.63	\	\
		Control	12	\	94.92 ± 12.21	807.83 ± 99.37	\	\
Hilt, 2000, Journal of endodontics, [11]	stainless steel K-type files (30 size 02 taper), torsional testing apparatus	10 cycles Statim autoclave	10	132 °C (270 °F), 20 psi, 30 min	98.9 ± 14.7	1704 ± 325	\	630 ± 18
		control	10	\	105.8 ± 4.9	1598 ± 226	\	616 ± 22
Canalda-Sahli, 1998, international endodontics, [19]	stainless steel flex of file (30 size 02 taper), Torquemeter Memocouple, Maillefer	10 cycles autoclave	10	136 °C, 2.2 bar, 10 min	61.70 ± 2.40	1306 ± 167.65	64.85 ± 3.28	\
		control	10	\	61.55 ± 3.28	1328 ± 188.46	63.30 ± 3.37	\
Haikel, 1997, international endodontics, [22]	flex of file (30 size 02 taper), torsional testing apparatus	10 cycles autoclave	10	180 °C 2.2 bar 2 h,	58.20 ± 2.06	1420. 6 ± 379	62.52 ± 2.19	\
		control	10	\	56.28 ± 5.36	1262.2 ± 293	59.60 ± 1.92	\

**Table 5:** Data extracted from studies concerning the torsional moments and the deflection angles of the instruments manufactured with the new alloys (M wire and controlled memory (CM) wire) at 0, 1, 2, 3, and 7 autoclave cycles.

Autor, Date, Journal, Reference Number	Endodontic Instruments, Test Instruments	Sterilization Methods	No. of Instruments	Temperature, Exposure Time	Torsional Moment Mean ± SD (g·cm)	Angular Deflection Mean ± SD (degrees)
King, 2011, international endodontic journal, [9]	Twisted file size 25, 0.06 taper Torsiometer/Memocouple	control	20	\	51.40 ± 6.46	586.55 ± 64.18
		Autoclave statim 1 cycles	20	132 °C for 6 min	46.32 ± 5.84	670.63 ± 59.74
		Autoclave statim 3 cycles	20	132 °C for 6 min,	43.25 ± 7.28	672.65 ± 103.16
		Autoclave statim 7 cycles	20	132 °C for 6 min,	48.70 ± 4.73	702..25 ± 80.64
	GT Series X M wire size 20, 0.06 taperTorsiometer/Memocouple	control	20	\	97.95 ± 11.04	445.65 ± 62.39
		Autoclave statim 1 cycles	20	132 °C for 6 min,	94.75 ± 8.84	441.80 ± 47.30
		Autoclave statim 3 cycles	20	132 °C for 6 min,	85.67 ± 9.59	483.11 ± 66.57
		Autoclave statim 7 cycles	20	132 °C for 6 min	78.10 ± 7.36	430.75 ± 66.49
Casper, R.B. 2011 journal of endodontic [10]	Profile Vortex m-wire microprocessor-controlled torsiometer	0	20	\	76 ± 7.0	438 ± 59
		1 cycles Autoclave statim	20	132 °C for 6 min	80 ± 9.9	441 ± 58.0
		2 cycles Autoclave statim	20	132 °C for 6 min	78 ± 8.3	448 ± 33.0
		3 cycles Autoclave statim	20	132 °C for 6 min	75 ± 4.3	441 ± 35.5
		7 cycles Autoclave statim	20	132 °C for 6 min	79 ± 8.2	452 ± 75.9
	Twisted Files microprocessor-controlled torsiometer	0	20, 16	\	28 ± 4.1	948 ± 110.8
		1 cycles Autoclave statim	20, 19	132 °C for 6 min	29 ± 2.7	916 ± 61.0
		2 cycles Autoclave statim	20, 19	132 °C for 6 min	29 ± 4.0	963 ± 147.0
		3 cycles Autoclave statim	20, 16	132 °C for 6 min	29 ± 4.9	898 ± 174.6
		7 cycles Autoclave statim	18, 18	132 °C for 6 min	28 ± 2.7	1075 ± 206.8
	File CM Wire microprocessor-controlled torsiometer	0	19, 20	\	87 ± 11.0	821 ± 34.7
		1 cycles Autoclave statim	20	132 °C for 6 min	91 ± 11.1	829 ± 56.4
		2 cycles Autoclave statim	19, 20	132 °C for 6 min	92 ± 11.2	820 ± 36.5
		3 cycles Autoclave statim	20	132 °C for 6 min	90 ± 9.1	815 ± 40.5
		7 cycles Autoclave statim	20, 18	132 °C for 6 min	87 ± 12.5	833 ± 89.8

**Table 6:** Assessment of risk of bias within the studies (Newcastle–Ottawa scale) with scores 7–12 = low quality, 13–20 = intermediate quality, and 21–24 = high quality.

		Selection			Comparability		Exposure		Score
Reference	Definition of Cases	Representativeness of Cases	Selection of Controls	Definition of Controls	Comparability of Cases and Controls on the Basis of the Design or Analysis	Ascertainment of Exposure	Same Method of Ascertainment for Cases and Controls	Non-Response Rate	
[11]	3	3	3	3	3	3	3	0	21
[18]	2	2	2	2	2	2	3	0	15
[19]	3	3	3	3	3	3	3	0	21
[9]	3	3	3	3	3	3	3	0	21
[10]	2	2	2	2	2	3	3	0	16
[20]	2	2	2	2	3	3	3	0	17
[21]	3	3	3	3	3	3	3	0	21
[22]	3	3	3	3	3	3	3	0	21
